# Care Partner Engagement in Secure Messaging Between Patients With Diabetes and Their Clinicians: Cohort Study

**DOI:** 10.2196/49491

**Published:** 2024-02-09

**Authors:** Wagahta Semere, Andrew J Karter, Courtney R Lyles, Mary E Reed, Leah Karliner, Celia Kaplan, Jennifer Y Liu, Jennifer Livaudais-Toman, Dean Schillinger

**Affiliations:** 1 Department of Medicine University of California San Francisco San Francisco, CA United States; 2 Center for Vulnerable Populations Zuckerberg San Francisco General Hospital San Francisco, CA United States; 3 Center for Aging in Diverse Communities University of California San Francisco San Francisco, CA United States; 4 Division of Research Kaiser Permanente Oakland, CA United States

**Keywords:** caregivers, diabetes, telehealth, secure messaging, patient portal, messaging, diabetes outcomes, family care, clinical care

## Abstract

**Background:**

Patient engagement with secure messaging (SM) via digital patient portals has been associated with improved diabetes outcomes, including increased patient satisfaction and better glycemic control. Yet, disparities in SM uptake exist among older patients and racial and ethnic underserved groups. Care partners (family members or friends) may provide a means for mitigating these disparities; however, it remains unclear whether and to what extent care partners might enhance SM use.

**Objective:**

We aim to examine whether SM use differs among older patients with diabetes based on the involvement of care partner proxies.

**Methods:**

This is a substudy of the ECLIPPSE (Employing Computational Linguistics to Improve Patient-Provider Secure Emails) project, a cohort study taking place in a large, fully integrated health care delivery system with an established digital patient portal serving over 4 million patients. Participants included patients with type 2 diabetes aged ≥50 years, newly registered on the patient portal, who sent ≥1 English-language message to their clinician between July 1, 2006, and December 31, 2015. Proxy SM was identified by having a registered proxy. To identify nonregistered proxies, a computational linguistics algorithm was applied to detect words and phrases more likely to appear in proxy messages compared to patient-authored messages. The primary outcome was the annual volume of secure messages (sent or received); secondary outcomes were the length of time to the first SM sent by patient or proxy and the number of annual SM exchanges (unique message topics generating ≥1 reply).

**Results:**

The mean age of the cohort (N=7659) at this study’s start was 61 (SD 7.16) years; 75% (n=5573) were married, 15% (n=1089) identified as Black, 10% (n=747) Chinese, 12% (n=905) Filipino, 13% (n=999) Latino, and 30% (n=2225) White. Further, 49% (n=3782) of patients used a proxy to some extent. Compared to nonproxy users, proxy users were older (*P*<.001), had lower educational attainment (*P*<.001), and had more comorbidities (*P*<.001). Adjusting for patient sociodemographic and clinical characteristics, proxy users had greater annual SM volume (20.7, 95% CI 20.2-21.2 vs 10.9, 95% CI 10.7-11.2; *P*<.001), shorter time to SM initiation (hazard ratio vs nonusers: 1.30, 95% CI 1.24-1.37; *P*<.001), and more annual SM exchanges (6.0, 95% CI 5.8-6.1 vs 2.9, 95% CI 2.9-3.0, *P*<.001). Differences in SM engagement by proxy status were similar across patient levels of education, and racial and ethnic groups.

**Conclusions:**

Among a cohort of older patients with diabetes, proxy SM involvement was independently associated with earlier initiation and increased intensity of messaging, although it did not appear to mitigate existing disparities in SM. These findings suggest care partners can enhance patient-clinician telecommunication in diabetes care. Future studies should examine the effect of care partners’ SM involvement on diabetes-related quality of care and clinical outcomes.

## Introduction

Patient portals are digital platforms that allow patients to securely access their personal health information, request prescription refills, schedule appointments, and communicate with their health care providers [1,2]. Driven in large part by federal meaningful use incentives, portal adoption by health care organizations has accelerated over the past decade [3]. Currently, over 90% of health care organizations offer patient portal access to their patients [4]. Social distancing measures during the COVID-19 pandemic led to restrictions on in-person visits and a dramatic shift to telehealth, making portal platforms and secure messaging (SM) increasingly relevant [1,5]. For patients with chronic diseases, such as diabetes, that rely upon regular intervisit communication with providers to support self-management, patient portals and SM can be critical to ensuring the provision of high-quality care. For example, patients with diabetes depend on communication with their providers to make timely and ongoing adjustments to their medications to avoid adverse events such as hypoglycemia and hyperglycemia [6]. Portal platforms and SM specifically can support this decision-making through asynchronous patient-provider communication. A recent systematic review highlighted significant associations between portal use and increased preventative behaviors, patient satisfaction, and medication adherence [7]. Among patients with diabetes, portal engagement has been associated with better medication adherence and self-efficacy, and SM use has been associated with better glycemic control [8-11].

Yet, many patients with medical and social vulnerabilities who may stand to benefit most from portal and SM use experience barriers to engagement. Several studies have documented substantial disparities in portal use among patients who are older, from diverse racial and ethnic backgrounds, and have lower educational attainment or limited health literacy [12-15]. Despite significant health system investment in patient portals, a recent national study found that only 15%-30% of patients offered portal access logged on [16]. Recent work has found that the reasons patients do not engage with portals likely extend beyond having limited access to technology infrastructure (computers and internet) to portal design features that limit broad accessibility [17]. Prominent features in the design of many patient portals include small font, English-only text, and complex user interfaces that limit access for patients with limited English proficiency, low health literacy, and disabilities [18].

Patients with lower health literacy and limited computer abilities who do manage to access the portal, experience less patient satisfaction than those with higher health literacy and computer abilities [19]. For these patients, care partners (family members or friends who assist patients with their health care needs, including communication) serving as proxies may offer a promising means for increasing portal engagement and accessing the potential benefits of SM. According to national survey data, one-third of caregivers use portals for their caregiving duties and are more likely to do so if they are caring for someone with a chronic condition [20]. Currently, care partners can access the patient portal and message clinicians in one of two ways: (1) *formally*, when a patient designates a registered proxy, who then has their own, linked account, and (2) *informally*, when a proxy logs on as the patient. Prior studies suggest that up to 18% of patient portal users share access with a care partner and anywhere from 25% to 50% of care partners report accessing the portal informally using the patient’s account [21,22]. The large proportion of proxies accessing the portal informally using patient credentials is likely due to the inconsistency with which health systems provide care partners portal access and the barriers that exist to registration and use [23]. However, these studies have relied on patient and caregiver self-reported use; fears of reporting unauthorized portal access may lead to an underestimate of actual use.

It is unclear how proxy involvement might influence patients’ SM engagement. Understanding the prevalence and characteristics of proxy messaging on behalf of patients is particularly important to inform the provision of patient care for diverse, aging populations. In this study, we leverage a novel computational linguistics algorithm to identify informal proxy involvement in SM among a cohort of older, racially and ethnically diverse patients with type 2 diabetes receiving care in a large, fully integrated health care delivery system with a mature patient portal. We follow this cohort over the course of 10 years, examining all secure messages patients exchanged with their clinicians. The objective of this study is to examine whether SM use varies based on care partner proxy involvement. We hypothesize that the involvement of proxies in SM is associated with increased SM communication and earlier initiation of messaging.

## Methods

### Study Sample and Setting

This is a substudy of the ECLIPPSE (Employing Computational Linguistics to Improve Patient-Provider Secure Emails) project, which leverages a large data set of secure messages exchanged between a cohort of patients with diabetes and their clinicians to understand the impact of patient health literacy and provider linguistic complexity on diabetes outcomes [24]. The ECLIPPSE cohort was drawn from the Diabetes Study of Northern California (DISTANCE). DISTANCE surveyed a racially or ethnically stratified (African American [n=6781, 17%], Asian [n=11,197, 27%], Latino/a/x/Hispanic hereafter referred to as Latino [n=7018, 17%], and White [n=4233, 10%]) random sample of patients with diabetes receiving care within Kaiser Permanente Northern California, a large, fully integrated health care delivery system serving over 4 million members in Northern California. In total, 20,188 patients with diabetes completed the survey—fielded in 2005-2006 using a combination of phone, computer, and paper distribution methods—designed to examine social and behavioral factors associated with disparities in diabetes-related care and outcomes [25]. ECLIPPSE included the subset of DISTANCE survey respondents who sent at least 1 secure message to their clinician in over a 10-year period (July 1, 2006, to December 31, 2015).

Kaiser Permanente Northern California launched its patient portal in 1999 and by late 2005, the portal allowed patients to securely exchange messages with providers. In 2006, the portal “Act for a Family Member” feature was activated, which allowed patients to formally designate a proxy (spouse, adult child, friend, or other care partner) to access the portal and send secure messages on their behalf. Outside of “Act for a Family Member,” it is not known how often proxy users access the portal and informally perform tasks on behalf of patients without registering as proxies. For this study, we included all patients in the ECLIPPSE cohort who were aged 50 years or older at the start of the observation period (July 1, 2006). We restricted the sample to those who composed English-language messages as the portal was only available in English at the start of this study’s period.

### Ethical Considerations

The University of California San Francisco and Kaiser Permanente Northern California institutional review boards approved this study (IRB#10-00671). Secondary analysis was permitted without additional consent. All study data were kept secure on password-protected servers to protect the privacy and confidentiality of the patient, care partner, and clinician.

### Development and Validation of the ProxyID Algorithm

In addition to formally registered proxies, we also identified those patients who were likely using informal proxies to communicate with providers via SM. We did this by applying *ProxyID*, an algorithm that uses computational linguistics to detect words and phrases more likely to appear in proxy SM compared to patient-authored SM. The development and validation of *ProxyID* has been described in detail previously [26]. Briefly, to develop *ProxyID,* proxy-authored SM written by registered proxy users were identified, then an equal number of presumed patient-authored SM were randomly sampled. Wordsmith Tools 6 was used to identify key n-grams (ie, words and contiguous phrases) significantly more likely than chance to occur in registered proxy SM compared to presumed patient-authored SM [27,28]. Examples of key n-grams included third-person pronouns and phrases such as “I am writing on behalf of.” The key n-grams for each secure message were fed into *ProxyID* which, through machine learning, selected likely proxy messages based on these data and patterns of n-grams in the messages. This ultimately enabled the classification of each secure message as likely proxy-authored versus likely patient-authored. To validate these classifications, 3 blinded expert assessors read secure messages from a purposive sample of 200 unique patients (100 secure messages designated by *ProxyID* as likely proxy-authored and 100 designated as likely patient-authored SM) and, based on SM content, categorized these secure messages as proxy-authored or patient-authored. *ProxyID* had moderate agreement with blinded expert categorization (κ=0.58), with a sensitivity of 0.93 (negative predictive value 0.95) and specificity of 0.70 (positive predictive value 0.64). Given the small number of registered proxies compared to informal proxies (see Results, below) identified by *ProxyID*, we grouped registered and informal proxies together for all analyses.

### Patient Sociodemographic and Clinical Characteristics

Patients’ self-reported sociodemographic characteristics (age, gender, race or ethnicity, marital status, and educational attainment) were obtained via the DISTANCE survey. The patient’s most recent hemoglobin A_1c_ (HbA_1c_) and Charlson comorbidity score before the survey receipt date were derived from the electronic health record [29]. Health care usage (outpatient, inpatient, and emergency room visits) over the 12 months before the survey receipt date was derived from the electronic health record.

### SM Characteristics

We examined SM characteristics during active SM use. We defined active SM use as starting from the time at which the patient first sent a secure message to the end of this study’s period; we censored due to patient disenrollment from the health plan or death. We defined our primary outcome, secure message volume, as the average secure message count per year during active SM use. We defined our secondary outcomes as (1) initiation: time to first patient-sent secure message from study start and (2) exchanges: average number of unique SM subjects generating ≥1 reply per year during active SM use.

### Statistical Analysis

*ProxyID* was applied to all secure messages sent by each patient to determine which patients had secure messages likely authored by a proxy. Patients with registered proxy-authored secure messages and those found to have one or more secure messages predicted by *ProxyID* to be proxy-authored during this study’s period were categorized as “any proxy.” Patients without proxy-authored messages over this study’s period were categorized as “never proxy.” The sociodemographic and clinical differences between “any proxy” versus “never proxy” patients were characterized using bivariate analyses; categorical values were reported as percentages and the Pearson chi-squared test was used to compare subgroups.

For annual SM volume and number of exchanges, we calculated person-years of observation for each patient during their period as active SM users. In a given year, only SM data from active SM users were included. We excluded SM data from patients who disenrolled from the health plan or died. Multivariable negative binomial regression models were specified to examine the association of patient proxy use with the average annual SM volume and number of exchanges. We selected the negative binomial regression as it provided the best fit for modeling count variables that are widely dispersed. The models accounted for repeated measures by patients (eg, some patients contributed up to 10 observations, one for each year of this study). Models were adjusted for patient sociodemographic (age, gender, race or ethnicity, marital status, educational attainment, and limited English proficiency status) and clinical (HbA_1c_, comorbidities, outpatient visits, emergency department visits, and hospital admissions) characteristics, as well as proxy use and year of messaging. A Cox proportional hazards regression model adjusted for the same patient sociodemographic and clinical characteristics used in the multivariable negative binomial models above, and proxy use was specified to simultaneously assess the effect of proxy use (reference: no use) on time (in days) to initiation of the first secure message. Model hazard ratios (HRs) of >1 indicated that proxy use was associated with a shorter time to initiation of messaging; HR<1 indicated proxy use was associated with a longer time to initiation of messaging. As all patients sent at least 1 message during this study’s period, no observations were censored for this analysis.

We examined whether the relationship between proxy status and SM volume differed by select patient characteristics, by adding interaction terms (proxy status × patient race or ethnicity and proxy status × educational attainment) to the adjusted multivariable regression models.

Statistical significance was defined as 2-tailed *P*<.05. All statistical analyses were performed using Stata (version 16.1; StataCorp).

## Results

### Cohort Characteristics

In total, 7659 patients met this study’s inclusion criteria. The mean age was 61 (SD 7.16) years at baseline, 46% (n=3548) were women, and the majority were married or partnered (75%). Patients self-identified as Black (n=1089, 15%), Chinese (n=747, 10%), Filipino (n=905, 12%), Latino (n=999, 13%), of other races or multiracial (n=817, 11%), and White or non-Hispanic (n=2225, 30%; Table 1). The person-time of observation among active SM users over this study’s period was 45,712 person-years (70,812 person-months; Multimedia Appendix 1)

**Table 1:** Characteristics of patients with type 2 diabetes by proxy engagement over the entire cohort study period, from 2006 to 2015 (N=7659)^a^.

Patient characteristics		Total (N=7659), n (%)	Never proxy (n=3877), n (%)	Any proxy (n=3782), n (%)	*P* value	
**Age (years)**						<.001
	50-59	3483 (45.5)	1933 (49.9)	1550 (41)		
	60-69	2877 (37.6)	1473 (38)	1404 (37.1)		
	70-79	1299 (16.9)	471 (12.1)	828 (21.9)		
Women		3548 (46.3)	1752 (45.2)	1796 (47.5)	.04	
**Race**						<.001
	Black	1089 (14.6)	587 (15.6)	502 (13.6)		
	Chinese	747 (10)	375 (10)	372 (10.1)		
	Filipino	905 (12.2)	506 (13.4)	399 (10.8)		
	Latino^b^	999 (13.4)	468 (12.4)	531 (14.4)		
	Other Asian	663 (8.9)	368 (9.8)	295 (8)		
	Other or mixed	817 (11)	388 (10.3)	429 (11.7)		
	White	2225 (29.9)	1073 (28.5)	1152 (31.3)		
Married or living with a partner		5573 (75.0)	2838 (75.5)	2735 (74.4)	.28	
**Education**						<.001
	Less than high school degree	861 (11.4)	343 (9)	518 (13.9)		
	High school	1911 (25.3)	888 (23.3)	1023 (27.5)		
	Some college or more	4768 (63.2)	2587 (67.8)	2181 (58.6)		
LEP^c,d^		499 (6.5)	194 (5)	305 (8.1)	<.001	
HbA_1c_^e^ ≥8%^f^		1705 (22.3)	871 (22.5)	834 (22.1)	.66	
**Charlson comorbidity^g^**						<.001
	1	4075 (53.2)	2225 (57.4)	1850 (48.9)		
	2	2152 (28.1)	1011 (26.1)	1141 (30.2)		
	3+	1432 (18.7)	641 (16.5)	791 (20.9)		
≥3 outpatient visits^g^		6467 (84.4)	3192 (82.3)	3275 (86.6)	<.001	
≥1 emergency department visit^g^		1471 (19.2)	682 (17.6)	789 (20.9)	<.001	
≥1 hospital admission^g^		701 (9.2)	315 (8.1)	386 (10.2)	.002	

^a^Percentages based on nonmissing values. Missing responses: race or ethnicity (n=214, 2.8%), marital status (n=227, 3%), education (n=119, 1.6%), and limited English proficiency (n=22, 0.3%).

^b^Includes Latino/a/x/Hispanic individuals.

^c^LEP: limited English proficiency.

^d^Respondents were asked, “How often do you have difficulty understanding or speaking English?” Responses were dichotomized as limited English proficiency (“Always,” “Often,” and “Sometimes”) and English proficient (“Rarely” and “Never”).

^e^HbA_1c_: hemoglobin A_1c_.

^f^Measured closest to study onset.

^g^Usage in the 12 months before this study’s entry.

### Patient Characteristics by Proxy Status

In total, 49% (n=3782) of patients were categorized as “any proxy” users; 95% (n=3585) were nonregistered proxies, while only 5% (n=197) were registered (Multimedia Appendix 2). In bivariate comparisons, “any proxy” users, when compared to “never proxy” users, were older (aged 70-79 years; 21.9%, n=828 vs 12.1%, n=471; *P*<.001), more likely to be women (47.5%, n=1796 vs 45.2%, n=1752; *P*=.04), have lower educational attainment (less than high school degree, 13.9%, n=518 vs 9%, n=343; *P*<.001), and have limited English proficiency (8.1%, n=305 vs 5%, n=194; *P*<.001). At baseline, “any proxy” users were more likely to have a mean Charlson comorbidity index greater than 3 (20.9%, n=791 vs 16.5%, n=641; *P*<.001) and more frequent health care usage in the 12 months before survey receipt, including outpatient (≥3 visits, 86.6%, n=3275 vs 82.3%, n=3192; *P*<.001), emergency department (≥1 visit, 20.9%, n=789] vs 17.6%, n=682; *P*<.001), and hospital (≥1 admission, 10.2%, n=386 vs 8.1%, n=315; *P*=.002; Table 1).

### SM Patterns by Proxy Status

In unadjusted models, “any proxy” users had nearly twice the volume of secure messages per year compared to “never proxy” users (21.3, 95% CI 20.8-21.8 vs 11.0, 95% CI 10.7-11.3; *P*<.001; Table 2) and double the SM exchanges per year (6.0, 95% CI 5.9-6.2 vs 3.0, 95% CI 2.9-3.0; *P*<.001). These findings were essentially unaltered by adjustment (volume of secure messages per year with any proxy use: 20.7, 95% CI 20.2-21.2 vs never proxy: 10.9, 95% CI 10.7-11.2; *P*<.001); SM exchanges per year (any proxy use: 6.0, 95% CI 5.8-6.1 vs never proxy: 2.9, 95% CI 2.9-3.0; *P*<.001). Compared to “never proxy” users, “any proxy” users had earlier initiation of messaging (unadjusted HR 1.19, 95% CI 1.14-1.25; *P*<.001; adjusted HR 1.30, 95% CI 1.24-1.37; *P*<.001). The relationship between proxy use and annual SM volume did not differ across patient race and ethnicity (*P*=.80) and educational attainment (*P*=.39) over the entire cohort study period.

**Table 2:** Annual secure message volume by patient characteristics over the entire cohort study period, from 2006 to 2015^a^.

Characteristics			Unadjusted hazard ratio (95% CI)		*P* value		Adjusted^b^ hazard ratio (95% CI)	*P* value
Never proxy			11.0 (10.7-11.3)		Reference		10.9 (10.7-11.2)	Reference
Any proxy			21.3 (20.8-21.8)		<.001		20.7 (20.2-21.2)	<.001
**Age (years)**								
	50-59	17.0 (16.5-17.5)		Reference		15.9 (15.5-16.3)		Reference
	60-69	15.9 (15.4-16.4)		.002		14.8 (14.4-15.2)		<.001
	70-79	16.9 (16.0-17.7)		.78		15.7 (15.0-16.5)		<.001
**Gender**								
	Men	16.6 (16.1-17.1)		Reference		15.5 (15.2-15.9)		Reference
	Women	16.5 (16.1-17.0)		.87		15.3 (15.0-15.7)		.85
**Race or ethnicity**								
	Black	16.7 (15.8-17.5)		.02		15.6 (14.8-16.4)		.002
	Chinese	15.4 (14.5-16.4)		<.001		14.5 (13.7-15.3)		.01
	Filipino	14.9 (14.0-15.7)		<.001		14.0 (13.3-14.7)		<.001
	Latino	16.1 (15.2-17.0)		.001		14.9 (14.2-15.6)		<.001
	Other Asian	15.8 (14.9-16.8)		<.001		14.8 (14.0-15.5)		.009
	Other or mixed	16.4 (15.4-17.3)		.005		15.2 (14.4-15.9)		<.001
	White	18.0 (17.4-18.7)		Reference		16.8 (16.3-17.3)		Reference
**Marital status**								
	Married or living with partner	16.4 (16.0-16.8)		Reference		15.2 (14.9-15.5)		Reference
	Never married or widowed or divorced	17.2 (16.6-17.9)		.03		16.3 (15.7-16.9)		.02
**Education**								
	Less than high school	15.9 (15.0-16.8)		Reference		15.1 (14.3-16.0)		Reference
	High school	16.4 (15.8-17.1)		.34		15.4 (14.9-15.9)		.91
	Some college or more	16.7 (16.3-17.1)		.11		15.5 (15.2-15.8)		.02
**English proficiency**								
	English proficient	16.7 (16.4-17.1)		Reference		15.6 (15.3-15.9)		Reference
	LEP^c,d^	13.3 (12.3-14.4)		<.001		12.7 (11.8-13.6)		<.001
**HbA_1c_^e,f^**								
	<8%	16.3 (16.0-16.7)		Reference		15.3 (15.0-15.6)		Reference
	≥8%	17.4 (16.7-18.1)		.008		16.1 (15.6-16.7)		.02
**Charlson comorbidities^f^**								
	1	15.3 (14.9-15.7)		Reference		14.3 (14.0-14.7)		Reference
	2	17.2 (16.5-17.8)		<.001		16.1 (15.6-16.6)		.003
	3+	19.4 (18.5-20.2)		<.001		18.1 (17.4-18.8)		<.001
**Number of outpatient visits^g^**								
	<3	13.8 (13.1-14.5)		Reference		13.0 (12.4-13.5)		Reference
	≥3	17.1 (16.7-17.4)		<.001		15.9 (15.6-16.2)		<.001
**Number of emergency department visits^g^**								
	None	16.1 (15.8-16.5)		Reference		15.0 (14.8-15.3)		Reference
	Any	18.5 (17.7-19.3)		<.001		17.3 (16.7-18.0)		.02
**Number of hospital admissions^g^**								
	None	16.4 (16.0-16.7)		Reference		15.3 (15.0-15.5)		Reference
	Any	18.5 (17.3-19.6)		<.001		17.4 (16.4-18.3)		.83

^a^Secure message volume: count of annual patient messages sent and received.

^b^Adjusted for age, sex, race or ethnicity, education, marital status, limited English proficiency status, hemoglobin A_1c_, comorbidities, number of outpatient visits, number of emergency department visits, number of hospital admissions, year of messaging, and proxy use.

^c^LEP: limited English proficiency.

^d^Respondents were asked, “How often do you have difficulty understanding or speaking English?” Responses were dichotomized as limited English proficiency (“Always,” “Often,” and “Sometimes”) and English proficient (“Rarely” and “Never”).

^e^HbA_1c_: hemoglobin A_1c_.

^f^Measured closest to study onset.

^g^Usage in the 12 months before this study’s entry.

### SM Patterns by Patient Sociodemographic and Clinical Characteristics

In adjusted multivariable models, patients unmarried or not living with a partner versus married or living with a partner sent and received more messages per year (16.3, 95% CI 15.7-16.9 vs 15.2, 95% CI 14.9-15.5; *P*=.02). Patients with limited English proficiency, compared to those who were English proficient, sent and received fewer messages annually (12.7, 95% CI 11.8-13.6 vs 15.6, 95% CI 15.3-15.9; *P*<.001). Patients with higher baseline HbA_1c_ had greater annual SM volume (16.1, 95% CI 15.6-16.7 vs 15.3, 95% CI 15.0-15.6, *P*<.001). More frequent health care usage in the 12 months before the survey receipt was associated with greater annual SM volume: having ≥3 outpatient visits (15.9, 95% CI 15.6-16.2 vs 13.0, 95% CI 12.4-13.5; *P*<.001) and any emergency department visits (17.3, 95% CI 16.7, 18.0 vs 15.0, 95% CI 14.8, 15.3; *P*=.02; Table 2).

## Discussion

### Principal Findings

SM is an increasingly important mode of communication in patient care and may have particular relevance for aging patients with chronic illnesses. Such patients often require additional support and can benefit from frequent digital communication for disease management [30-32]. Yet, little is known about how care partners access secure messages on patients’ behalf. Among a racially and ethnically diverse older cohort of patients with diabetes, those patients involving proxies in messaging had a greater annual volume of messages, earlier initiation of messaging as well as more message exchanges with their clinicians. However, while involving proxies increased messaging overall, it did not appear to mitigate existing race or ethnic disparities in SM use.

Care partners have key roles in providing support for patients with chronic diseases by taking on responsibilities including coordinating health care tasks, accompanying patients to medical visits, and communicating with clinicians [32,33]. Prior studies suggest that care partners participate in primary care visits for nearly 40% of older adults with chronic illnesses, engaging in conversations and care decisions [34,35]. Given the increasing uptake of telehealth, more of these visit-based conversations are likely to occur remotely and digitally, leveraging platforms such as patient portals. We estimated that nearly half of patients with diabetes in our sample engaged proxies, which is higher than prior estimates [21]. This may be due to this study’s health system having a mature patient portal with an early investment in supporting design features, such as ease of use across mobile platforms and a focus on digital accessibility for those with disabilities that allow for wider accessibility for both proxies and patients. Despite having a process for formal proxy registration (“Act for a Family Member”), only 5.2% (n=197) of proxies in our sample were formally registered with the majority, identified using *ProxyID*, likely accessing the portal informally. This suggests that additional exploration is needed to understand design changes that may facilitate proxy registration. Other studies report that 25%-50% of proxies use portals without formally registering [21,22]. These prior estimates rely on self-report and may reflect a reluctance to disclose unauthorized use, thus underestimating rates of informal proxy use. A more recent smaller study focused on dementia care that employed a manual review of message authorship found that care partners overwhelmingly (97%) used patient credentials to access the portal [36]. Prior studies have not focused on large study samples or patients with diabetes, who have self-management support needs that may indicate a reliance on proxies. Designing portals and SM to be easily accessible to all users, can help ensure these communication platforms support patient- and family-centered care.

Patients engaging care partners as proxies were more likely to be older, have less educational attainment, and have limited English proficiency. This is not surprising given the well-documented challenges that older patients and those with communication barriers face in accessing and engaging with health care technology [37,38]. Care partners may be able to support SM engagement for patients who experience barriers to use. Women were more likely to have proxy SM involvement, which may be reflective of women being more likely than men to have a child or child-in-law provide care as opposed to a spouse [39]; younger rather than older generation care partners are more comfortable using technology to support their roles providing care [40]. Patients with more comorbidities and more frequent health care usage, suggestive of more complex care needs, were also more likely to engage proxies. This finding is consistent with prior work demonstrating that care partner use of technology for health care–related activities is more common when more intensive support is needed [41].

Patients who engaged proxies demonstrated greater SM engagement across several metrics. First, proxy-engaging patients initiated messaging earlier than those without proxy involvement. While it is not clear whether proxies specifically initiated messaging, our findings suggest that care partners assisted patients in the uptake and adoption of SM. Second, patients with proxies had a higher annual volume of messages and number of exchanges with their clinicians. These results are consistent with prior research suggesting that care partners are interested in leveraging health technology to support their loved ones and care-related activities [41]. Importantly, involving a proxy was associated with similar increases in the volume of messaging across patient racial and ethnic groups and levels of educational attainment. This suggests that proxy involvement may enable patient populations who experience barriers to engagement to reap the benefits of this remote technology.

Our study has important limitations. First, we identified patients who engage proxies using a novel computational linguistics algorithm, *ProxyID*, that has been validated in 1 health system. While *ProxyID* has demonstrated high sensitivity in excluding nonproxy messages, its lower specificity suggests that we likely misclassified some patient-authored messages as proxy-authored. This may have led to an overestimation of the number of patients using proxies. Conversely, some “hidden” proxies may have avoided language in secure messages that *ProxyID* could identify, thus leading to an underestimation of proxy engagement. However, the presence of hidden proxies in the sample designated as never proxy users would introduce a conservative bias (ie, underestimation of differences) in our assessment comparing those identified as proxy users versus never proxy users. Patients considered proxy users had varying degrees of proxy engagement in messaging that may have been associated with differences in SM patterns. Additionally, we are reporting data from 1 health system, limiting generalizability. This study’s setting, however, represents a large integrated health care system with advanced and frequent portal use. The sample was socioeconomically and ethnically diverse, except excluding the extremes of income [25]. Study data were gathered before the COVID-19 pandemic, which has been associated with an increase in SM across health systems including within our study setting [42]. Given the large, detailed nature of this study’s data and that the health system was an early adopter of the patient portal, the data set provides a unique opportunity to comprehensively examine broad patient SM patterns and the understudied area of proxy engagement. However, our findings may not reflect current SM patterns. Finally, our study design did not include analyses of SM content, or exploration of how proxy involvement might influence SM content or alter patient care.

### Conclusion

To our knowledge, this is the first study to describe how proxy involvement influences engagement with SM for older patients with diabetes. Proxy use was prevalent, with about half of patients engaging proxies to some extent. Proxy engagement was associated with earlier initiation of messaging, a greater volume of messages, and more exchanges with clinicians. Patients engaging proxies represented a more socially and medically vulnerable group. The benefits of proxy involvement were similar across patient race and ethnicity and across levels of educational attainment, thus unlikely to mitigate existing disparities in SM use. These findings suggest that engaging proxies may provide a pathway to increase SM uptake for patients with barriers to use, enabling access to its potential benefits. Modifying portal privacy and security rules may better accommodate proxy portal use on behalf of patients. Future work should explore avenues for identifying patients who may benefit from engaging proxies and determining if proxy involvement in messaging influences patient and care partner outcomes.

## Appendix

Multimedia Appendix 1 Total person-time of observation among patients with type 2 diabetes who are active users over the entire cohort study period, from 2006-2015 (N=7,659 patients). Active users were defined by starting observation from patient/proxy initiation of first secure message to the end of the study period or to patient leaving the health system if the patient left before the end of the study period.

Multimedia Appendix 2 Type of portal access and proxy authorship for any proxy users on behalf of patients with type 2 diabetes over the entire cohort study period, from 2006-2015 (N=3,782).

## Abbreviations

DISTANCE: Diabetes Study of Northern California
ECLIPPSE: Employing Computational Linguistics to Improve Patient-Provider Secure Emails
HbA_1c_: hemoglobin A_1c_
HR: hazard ratio
SM: secure messaging

## Data Availability

The data sets generated or analyzed during this study are not publicly available due to the need to maintain strict protection of patient and care partner privacy.

## References

[1] Patel PD, Cobb J, Wright D, Turer R, Jordan T, Humphrey A, Kepner AL, Smith G, Rosenbloom ST (2020). Rapid development of telehealth capabilities within pediatric patient portal infrastructure for COVID-19 care: barriers, solutions, results. J Am Med Inform Assoc.

[2] The ONC patient engagement playbook. The Office of the National Coordinator for Health Information Technology (ONC).

[3] Washington V, DeSalvo K, Mostashari F, Blumenthal D (2017). The HITECH Era and the path forward. N Engl J Med.

[4] Heath S (2020). Patient portal adoption tops 90%, but strong patient use is needed. Patient Engagement HIT.

[5] Judson TJ, Odisho AY, Neinstein AB, Chao J, Williams A, Miller C, Moriarty T, Gleason N, Intinarelli G, Gonzales R (2020). Rapid design and implementation of an integrated patient self-triage and self-scheduling tool for COVID-19. J Am Med Inform Assoc.

[6] American Diabetes Association Professional Practice Committee (2022). 6. Glycemic targets: standards of medical care in diabetes-2022. Diabetes Care.

[7] Carini E, Villani L, Pezzullo AM, Gentili A, Barbara A, Ricciardi W, Boccia S (2021). The impact of digital patient portals on health outcomes, system efficiency, and patient attitudes: updated systematic literature review. J Med Internet Res.

[8] Harris LT, Haneuse SJ, Martin DP, Ralston JD (2009). Diabetes quality of care and outpatient utilization associated with electronic patient-provider messaging: a cross-sectional analysis. Diabetes Care.

[9] Harris LT, Koepsell TD, Haneuse SJ, Martin DP, Ralston JD (2013). Glycemic control associated with secure patient-provider messaging within a shared electronic medical record: a longitudinal analysis. Diabetes Care.

[10] Wade-Vuturo AE, Mayberry LS, Osborn CY (2013). Secure messaging and diabetes management: experiences and perspectives of patient portal users. J Am Med Inform Assoc.

[11] Graetz I, Huang J, Muelly ER, Fireman B, Hsu J, Reed ME (2020). Association of mobile patient portal access with diabetes medication adherence and glycemic levels among adults with diabetes. JAMA Netw Open.

[12] Sarkar U, Karter AJ, Liu JY, Adler NE, Nguyen R, López A, Schillinger D (2011). Social disparities in internet patient portal use in diabetes: evidence that the digital divide extends beyond access. J Am Med Inform Assoc.

[13] Goel MS, Brown TL, Williams A, Hasnain-Wynia R, Thompson JA, Baker DW (2011). Disparities in enrollment and use of an electronic patient portal. J Gen Intern Med.

[14] Wallace LS, Angier H, Huguet N, Gaudino JA, Krist A, Dearing M, Killerby M, Marino M, DeVoe JE (2016). Patterns of electronic portal use among vulnerable patients in a nationwide practice-based research network: from the OCHIN Practice-Based Research Network (PBRN). J Am Board Fam Med.

[15] Graetz I, Huang J, Brand RJ, Hsu J, Yamin CK, Reed ME (2018). Bridging the digital divide: mobile access to personal health records among patients with diabetes. Am J Manag Care.

[16] United States Government Accountability Office (GAO) (2017). Health information technology: HHS should assess the effectiveness of its efforts to enhance patient access to and use of electronic health information. GAO-17-305.

[17] Anthony DL, Campos-Castillo C, Lim PS (2018). Who isn't using patient portals and why? Evidence and implications from a national sample of US adults. Health Aff (Millwood).

[18] Lyles CR, Fruchterman J, Youdelman M, Schillinger D (2017). Legal, practical, and ethical considerations for making online patient portals accessible for all. Am J Public Health.

[19] Wong JIS, Steitz BD, Rosenbloom ST (2019). Characterizing the impact of health literacy, computer ability, patient demographics, and portal usage on patient satisfaction with a patient portal. JAMIA Open.

[20] Turner K, Wei G, Otto AK, Reblin M (2021). Caregivers' use of patient portals: findings from a 2019 national survey. J Gen Intern Med.

[21] Reed ME, Huang J, Brand R, Ballard D, Yamin C, Hsu J, Grant R (2018). Communicating through a patient portal to engage family care partners. JAMA Intern Med.

[22] Wolff JL, Berger A, Clarke D, Green JA, Stametz R, Yule C, Darer JD (2016). Patients, care partners, and shared access to the patient portal: online practices at an integrated health system. J Am Med Inform Assoc.

[23] Latulipe C, Mazumder SF, Wilson RKW, Talton JW, Bertoni AG, Quandt SA, Arcury TA, Miller DP (2020). Security and privacy risks associated with adult patient portal accounts in US hospitals. JAMA Intern Med.

[24] Schillinger D, McNamara D, Crossley S, Lyles C, Moffet HH, Sarkar U, Duran N, Allen J, Liu J, Oryn D, Ratanawongsa N, Karter AJ (2017). The next frontier in communication and the ECLIPPSE study: bridging the linguistic divide in secure messaging. J Diabetes Res.

[25] Moffet HH, Adler N, Schillinger D, Ahmed AT, Laraia B, Selby JV, Neugebauer R, Liu JY, Parker MM, Warton M, Karter AJ (2009). Cohort profile: the Diabetes Study of Northern California (DISTANCE)—objectives and design of a survey follow-up study of social health disparities in a managed care population. Int J Epidemiol.

[26] Semere W, Crossley S, Karter AJ, Lyles CR, Brown W, Reed M, McNamara DS, Liu JY, Schillinger D (2019). Secure messaging with physicians by proxies for patients with diabetes: findings from the ECLIPPSE study. J Gen Intern Med.

[27] Hunston S (2013). McEnery, T. and Hardie, A. 2012. Corpus linguistics: method, theory & practice. Int J Corpus Linguistics.

[28] Crossley SA, Allen LK, Kyle KK, McNamara DS (2014). Analyzing discourse processing using a simple natural language processing tool. Discourse Process.

[29] Charlson ME, Pompei P, Ales KL, MacKenzie CR (1987). A new method of classifying prognostic comorbidity in longitudinal studies: development and validation. J Chronic Dis.

[30] (2022). Chronic disease caregiving through a public health lens: the framework for family caregiving and public health. The National Alliance for Caregiving.

[31] Fields B, Makaroun L, Rodriguez KL, Robinson C, Forman J, Rosland A (2022). Caregiver role development in chronic disease: a qualitative study of informal caregiving for veterans with diabetes. Chronic Illn.

[32] Riffin C, Van Ness PH, Wolff JL, Fried T (2019). Multifactorial examination of caregiver burden in a national sample of family and unpaid caregivers. J Am Geriatr Soc.

[33] Riffin C, Van Ness PH, Wolff JL, Fried T (2017). Family and other unpaid caregivers and older adults with and without dementia and disability. J Am Geriatr Soc.

[34] Wolff JL, Roter DL (2011). Family presence in routine medical visits: a meta-analytical review. Soc Sci Med.

[35] Wolff JL, Roter DL (2008). Hidden in plain sight: medical visit companions as a resource for vulnerable older adults. Arch Intern Med.

[36] Gleason KT, Wu MMJ, Wec A, Powell DS, Zhang T, Gamper MJ, Green AR, Nothelle S, Amjad H, Wolff JL (2023). Use of the patient portal among older adults with diagnosed dementia and their care partners. Alzheimers Dement.

[37] Gordon NP, Hornbrook MC (2016). Differences in access to and preferences for using patient portals and other eHealth technologies based on race, ethnicity, and age: a database and survey study of seniors in a large health plan. J Med Internet Res.

[38] Walker DM, Hefner JL, Fareed N, Huerta TR, McAlearney AS (2020). Exploring the digital divide: age and race disparities in use of an inpatient portal. Telemed J E Health.

[39] Li W, Manuel DG, Isenberg SR, Tanuseputro P (2022). Caring for older men and women: whose caregivers are more distressed? A population-based retrospective cohort study. BMC Geriatr.

[40] (2022). U.S. Caregivers' use of technology. American Association of Retired Persons (AARP).

[41] Zulman DM, Piette JD, Jenchura EC, Asch SM, Rosland A (2013). Facilitating out-of-home caregiving through health information technology: survey of informal caregivers' current practices, interests, and perceived barriers. J Med Internet Res.

[42] Neeman E, Lyon L, Sun H, Conell C, Reed M, Kumar D, Kolevska T, Kotak D, Sundaresan T, Liu R (2022). Future of teleoncology: trends and disparities in telehealth and secure message utilization in the COVID-19 era. JCO Clin Cancer Inform.

