# Supplementary material for: Care Partner Engagement in Secure Messaging Between Patients With Diabetes and Their Clinicians: Cohort Study
Source: JMIR Diabetes. 2024 Feb 9;9:e49491. doi: 10.2196/49491 (PMC10891488; doi:10.2196/49491)
Supplement: Multimedia Appendix 1 [file diabetes_v9i1e49491_app1.pdf]

Supplement A. Total person-time of observation among patients with type 2 diabetes who are active users over the entire cohort study period, from 2006-2015 (N=7,659 patients). Active users were defined by starting observation from patient/proxy initiation of first secure message to the end of the study period or to patient leaving the health system if the patient left before the end of the study period.

Year	Total person-months of observation among “active users”	Total person-years of observation among “active users”
2006	11,477	956.4
2007	29,013	2,417.8
2008	42,372	3,531.0
2009	53,097	4,424.7
2010	61,118	5,093.2
2011	67,384	5,615.3
2012	70,024	5,835.3
2013	71,554	5,962.8
2014	71,694	5,974.5
2015	70,812	5,901.0
Entire study period	548,545 person-months	45,712 person-years
