# Supplementary material for: Care Partner Engagement in Secure Messaging Between Patients With Diabetes and Their Clinicians: Cohort Study
Source: JMIR Diabetes. 2024 Feb 9;9:e49491. doi: 10.2196/49491 (PMC10891488; doi:10.2196/49491)
Supplement: Multimedia Appendix 2 [file diabetes_v9i1e49491_app2.pdf]

Multimedia Appendix 2. Type of portal access and proxy authorship for any proxy users on behalf of patients with type 2 diabetes over the entire cohort study period, from 2006-2015 (N=3,782)

	No (%)
Type of proxy use	
Registered proxy use	197 (5.2)
Informal or “hidden” proxy use	3,585 (94.8)
Percent of all patient messages (2006 – 2015) authored by any proxy	
(continuous %) of secure messages by any proxy	27% ± 30% (range 0.4-100%)
<50% of secure messages by any proxy	3,007 (79.5)
≥50% of secure messages by any proxy	775 (20.5)
